# Photothermal Self-Excitation of a Phase-Controlled Microcantilever for Viscosity or Viscoelasticity Sensing

**DOI:** 10.3390/s22218421

**Published:** 2022-11-02

**Authors:** João Mouro, Paolo Paoletti, Marco Sartore, Massimo Vassalli, Bruno Tiribilli

**Affiliations:** 1 Institute for Complex Systems, National Research Council (ISC-CNR), 50019 Florence, Italy; 2 School of Engineering, University of Liverpool, Liverpool L69 3GH, UK; 3 Elbatech Srl, 57030 Marciana, Italy; 4 School of Engineering, University of Glasgow, Glasgow G12 8LT, UK

**Keywords:** Phase-Locked Loop, microcantilever, sensing, viscosity, viscoelasticity, elastic modulus, viscous modulus

## Abstract

This work presents a feedback closed-loop platform to be used for viscosity or viscoelasticity sensing of Newtonian or non-Newtonian fluids. The system consists of a photothermally excited microcantilever working in a digital Phase-Locked Loop, in which the phase between the excitation signal to the cantilever and the reference demodulating signals is chosen and imposed in the loop. General analytical models to describe the frequency and amplitude of oscillation of the cantilever immersed in viscous and viscoelastic fluids are derived and validated against experiments. In particular, the sensitivity of the sensor to variations of viscosity of Newtonian fluids, or to variations of elastic/viscous modulus of non-Newtonian fluids, are studied. Interestingly, it is demonstrated the possibility of controlling the sensitivity of the system to variations of these parameters by choosing the appropriate imposed phase in the loop. A working point with maximum sensitivity can be used for real-time detection of small changes of rheological parameters with low-noise and fast-transient response. Conversely, a working point with zero sensitivity to variations of rheological parameters can be potentially used to decouple the effect of simultaneous external factors acting on the resonator.

## 1. Introduction

Measuring the viscosity of a fluid or understanding the behaviour of complex fluids is critical for a wide range of applications in the Industrial and Medical sectors. Most of the synthetic and biological fluids with interest to the process industry, environmental monitoring, healthcare, and microfluidics, exhibit a complex behaviour as a result of a nonlinear time-dependent relation between shear stress and shear strain rate. However, basic knowledge of their properties and ways to characterise them is still insufficient. Current microrheology techniques rely on following the trajectories of microbeads immersed in the fluid, or, alternatively, the active manipulation of probes within the fluid using magnetic or optical tweezers. These methods tend to be computationally intensive, requiring dedicated equipment, video tracking, and statistical treatment of data [1,2,3]. 

An alternative strategy is to use a vibrating mechanical microdevice, whose dynamic response depends on the rheological properties of the surrounding media [4,5]. The interactions between the cantilever and the viscous fluid were analytically modelled by calculating the hydrodynamic force caused by the fluid flow around the out-of-plane flexural and torsional oscillations of a microcantilever [6,7]. The hydrodynamic force was then used to relate the phase and amplitude responses of the microcantilever with the viscosity and density of the fluid [8,9,10,11,12,13,14,15]. 

More recently, the analytical expressions of the hydrodynamic force were extended to include the properties of a viscoelastic fluid. The amplitude and phase responses of a cantilever immersed in a viscoelastic fluid can then be used to extract its elastic and viscous modulus, in a broad range of frequencies [16,17,18]. These methods are relatively easy to implement, require very short computational time and allow choosing the range of frequencies to probe by using different geometries or modes of the cantilever [19]. They can also measure local viscosities in smaller time and space scales, using minimal volumes of liquid. However, these are typically based on the external excitation of the cantilever (using a frequency sweep), and limited by the low quality factor of the oscillations in highly viscous media. In addition, the vibrations caused by most excitation techniques couple with the experimental apparatus to make the resonance peak almost indistinguishable (the so-called “forest of peaks” problem [20,21]). 

These limitations can be mitigated by inducing the self-oscillation of the resonator in closed-loop feedback platforms, with a better signal-to-noise ratio and quality factor. The problem with these strategies is that they tend to be very non-linear and difficult to model and analyse [22,23,24,25,26]. The use of a Phase-Locked Loop (PLL) to detect low-noise and real-time shifts of frequency caused by changes in the viscosity of the fluid has recently been successfully proposed [27].

In this work, we develop a digital Phase-Locked Loop (PLL) to induce self-oscillations of a microcantilever immersed in viscous or viscoelastic media. The cantilever is photothermally excited to obtain a clean and linear response [28,29], and avoid the problem of the “forest of peaks”. We then extend the existing formulations of the hydrodynamic force, both for viscous and viscoelastic fluids, to analytically describe the dynamical response of the developed closed-loop PLL platform, and validate these models on the basis of experimental data. A thorough study of the sensitivity of the system to different rheological parameters is performed, demonstrating the possibility of operating this platform in different sensing regimes, according to the desired application.

The PLL configuration makes it possible to obtain low-noise and real-time signals, with fast transient responses, and high signal-to-noise ratios, overcoming most of the limitations of the current technology based on open-loop configurations.

## 2. Materials and Methods

### 2.1. Experimental Setup

This section describes the closed-loop platform developed in this work. The system consists of a digital Phase-Locked Loop (PLL), which uses a Proportional–Integral (PI) controller to track the oscillation frequency of a microcantilever. The frequency and amplitude of oscillation depend on the phase between the excitation force and reference signals, imposed in the PLL by the user. Any environmental change can then be detected by measuring shifts in frequency and/or the amplitude of oscillation. 

#### 2.1.1. Optomechanical Block

The microcantilever oscillates inside a closed PEEK cell, immersed in a viscous fluid. Its deflection is photothermally excited using an intensity modulated blue laser (Cobolt 06-MDL 405 nm), focused on its top surface. The modulated intensity causes a periodic temperature change on a region of the cantilever, which then induces oscillation due to the different thermal expansion of the bilayer. The optimal excitation point is located in regions of maximum curvature [29], which, in the case of the fundamental resonance mode used in this work, corresponds to a point close to the clamp [28]. 

The excited deflection is optically detected with a red laser focused near the tip of the cantilever, on its bottom surface, and reflected to a four-quadrant detector. 

#### 2.1.2. Demodulating and Control Electronics

**Figure 1:**
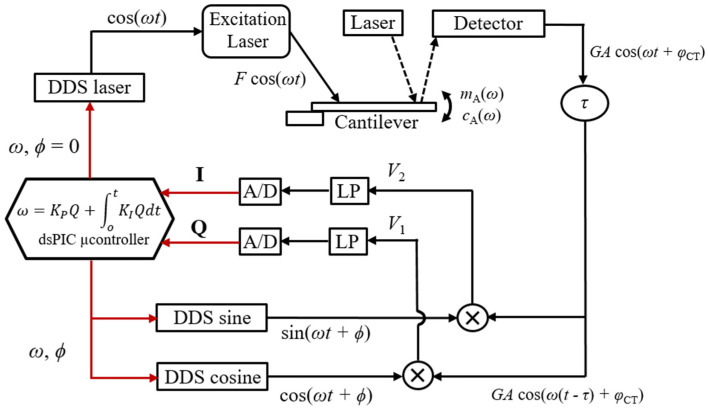
Schematic of the developed PLL platform.

The deflection signal coming from the four-quadrant detector is converted to a voltage and demodulated by two reference Direct-Digital-Synthesis signals, as shown in the schematic presented in Figure 1 (DDS-sine and DDS-cosine). The in-phase and quadrature components, *Q* and *I*, respectively, are filtered and converted to digital (A/D). The digital *Q*-signal is fed to a dsPIC microcontroller, and used as the error parameter in a programmed PI-microcontroller which continuously adjusts the frequency ω of the synthetised DDS reference signals and another DDS signal, used to excite the microcantilever (DDS-laser). The excitation signal (DDS-laser) and the reference signals (DDS-sine and DDS cosine) are generated with a phase ϕ between them, imposed by the user. The excitation signal (DDS-laser) at frequency ω is finally fed to the modulated blue laser, and used to excite the cantilever. 

To achieve high-resolution frequency generation, the clock frequency is set to 24 MHz, and the phase accumulators used in the DDSs contain 28 bits. With these parameters, the theoretical frequency resolution of the system is 24 × 10^6^/2^28^ = 0.0894 Hz.

#### 2.1.3. Electrical Scheme of the PLL

The electrical scheme of the PLL described in the previous sections is shown in Figure 1 and used to illustrate the functioning of the system.

### 2.2. General Modeling Equations of the PLL 

The frequency of oscillation of the PLL, ω, can be obtained by assuming that the microcontroller is perfectly tuned and that the steady state of the system works with an in-phase component *Q* = 0. This corresponds to solving the following condition:

(1) $$-\omega \tau +\mathrm{atan}\left(-\frac{\;\omega \left(c_{0}+c_{A}\right)}{m_{0}\omega_{0}^{2}-\left(m_{0}+m_{A}\right)\omega^{2}}\right)-\phi =-\left(\frac{\pi }{2}+n\pi \right),\mathrm{with}n=0,1,2\ldots ,$$

where ϕ is the imposed phase in the PLL, τ is the delay along the loop, mostly due to conversion of optical energy into mechanical energy in the photothermal actuation, $\omega_{0}$, $m_{0}$ and $c_{0}$ are the natural frequency in vacuum, total mass and intrinsic damping of the cantilever, respectively, and $m_{A}$ and $c_{A}$ are the added mass and damping terms due to the presence of the dissipative fluid.

The quadrature signal can be calculated using

(2) $$I=\frac{GA}{2}\sin\left(\frac{\pi }{2}+n\pi \right)=\{\begin{array}{c} \frac{GA}{2},\;for\;n=0,\;2,\;4,\ldots \\ -\frac{GA}{2},\;for\;n=1,\;3,\;5,\ldots \end{array},$$

where A is the amplitude of oscillation of the cantilever in the fluid and *G* is the transduction gain. Treating the microcantilever as a harmonic forced damped oscillator, its amplitude of oscillation is given by

(3) $$A_{norm}\left(\omega \right)=\frac{\left(m_{0}+m_{A}\right)}{{\left[{\left(m_{0}\omega_{0}^{2}-\omega^{2}m_{0}-\omega^{2}m_{A}\right)}^{2}+\omega^{2}{\left(c_{0}+c_{A}\right)}^{2}\right]}^{\frac{1}{2}}}$$

In this equation, $A_{norm}\left(\omega \right)$ is the amplitude of oscillation normalised with respect to the force applied to the cantilever and its mass, where $A_{norm}\left(\omega \right)=\frac{A\left(\omega \right)m_{0}G}{F}$. These general working expressions of the PLL are derived in detail in Part I of the Supplementary Materials. 

### 2.3. General PLL Model Applied to Purely Viscous Newtonian Fluids 

According to Sader [6] and Maali [30], the added mass and damping terms, $m_{A}$ and $c_{A}$, of a purely viscous fluid are given by

(4) $$m_{A}\left(\omega \right)=\frac{\pi }{4}\rho LW^{2}(a_{1}+\frac{a_{2}}{W}\sqrt{\frac{2\eta }{\rho \omega }}),$$

(5) $$c_{A}\left(\omega \right)=\frac{\pi }{4}\rho LW^{2}\omega \left(\frac{b_{1}}{W}\sqrt{\frac{2\eta }{\rho \omega }}+\frac{b_{2}}{W^{2}}\frac{2\eta }{\rho \omega }\right),$$

where ω is the oscillation frequency of the system, ρ and η are the density and viscosity of the fluid, *a*_1_ = 1.0553, *a*_2_ = 3.7997, *b*_1_ = 3.8018, and *b*_2_ = 2.7364 are constants to describe the hydrodynamic function [30], and L and W are the length and width of the microcantilever. These expressions are shown in Part I.1 of the Supplementary Materials. 

In the case of a purely viscous Newtonian fluid, Equations (4) and (5) are substituted into Equations (1) and (3) to obtain the frequency and amplitude of oscillation as function of the viscosity and density of the fluid. 

**Figure 2:**
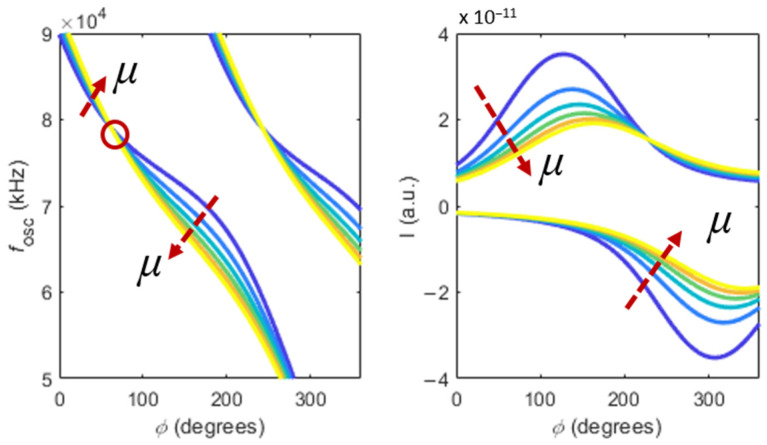
Frequencies of oscillation (**left**) and quadrature component of deflection *I* (**right**), as function of the imposed phase ϕ in the PLL and the viscosity of a Newtonian fluid. Two branches are observed for n=2 (**left branch**) and n=3 (**right branch**). Viscosity values range between $\eta =5\times 10^{-4}$ Pa s and $\eta =3\times 10^{-3}$ Pa s, in steps of $\Delta \eta =5\times 10^{-4}$ Pa s.

Figure 2 shows results of using this model to study the effect of changing the viscosity of the fluid, considering a fixed cantilever geometry (*L* = 150 µm, *W* = 28 µm, *T* = 3.0 µm, *f*_0_ = 164.98 kHz) and a fixed delay in the loop (τ=9.0 µs). In this simulation, the viscosity of the Newtonian liquid was swept between $\eta =5\times 10^{-4}$ Pa s and $\eta =3\times 10^{-3}$ Pa s, in steps of $\Delta \eta =5\times 10^{-4}$ Pa s. The density of the solution was assumed to be constant and independent of the viscosity, ρ=998 kg/m^3^ at 20 °C [23].

Two distinct branches can be observed in Figure 2. These correspond to different values of n in the simulations of Equations (1) and (2). Experimentally, these branches can be observed by inverting the gains of the PI-controller. However, the branches are merely a periodic repetition of the response of the cantilever, function of the imposed phase in the PLL, and describing a single branch is enough to understand the response of the system. 

As shown in Figure 2, increasing the viscosity causes a general decrease in both the frequency and amplitude of oscillation. However, for low values of imposed phase, the frequency seems to increase with the viscosity. The point highlighted in red shows a specific point of imposed phase in the loop whose frequency does not depend on the viscosity of the solution. 

### 2.4. General PLL Model Applied to Viscoelastic Non-Newtonian Fluids 

The added mass and damping terms, $m_{A}$ and $c_{A}$, of a viscoelastic fluid were first derived in [16,17,18] and are given by

(6) $$m_{A}\left(\omega \right)=C+D\frac{G^{\prime }}{\omega^{2}}+\frac{B}{\omega }\sqrt{\sqrt{{G^{\prime }}^{2}+{G^{\prime \prime }}^{2}}+G^{\prime }},$$

(7) $$c_{A}\left(\omega \right)=D\frac{G^{\prime \prime }}{\omega }+B\sqrt{\sqrt{{G^{\prime }}^{2}+{G^{\prime \prime }}^{2}}-G^{\prime }},$$

where $B=\frac{b_{1}\pi LW\sqrt{\rho }}{2\sqrt{2}}$, $C=\frac{\pi \rho LW^{2}}{4}$ and $D=\frac{\pi Lb_{2}}{2}$, and $G^{\prime }$ and $G^{\prime \prime }$ are the elastic and viscous modulus of the viscoelastic fluid, respectively [31]. These expressions are derived in detail in Part I.2 of the Supplementary Materials. 

**Figure 3:**
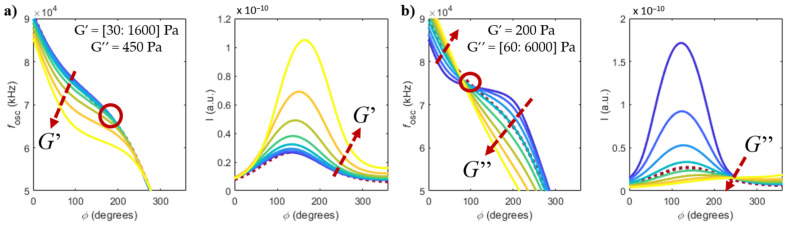
Frequencies of oscillation and quadrature component of deflection *I*, as a function of the imposed phase in the PLL and the (**a**) elastic modulus and (**b**) viscous modulus of a viscoelastic fluid. The dashed burgundy line represents the case of purely viscous water. Only the branch with *n* = 2 is shown.

In the case of a viscoelastic fluid, Equations (6) and (7) are then substituted into Equations (1) and (3) to obtain the frequency and amplitude of oscillation as function of the elastic and viscous modulus of the viscoelastic Maxwell fluid (for constant density). Figure 3a,b show the results of using this model to study the effect of changing the elastic and viscous modulus of the viscoelastic fluid, respectively, considering the same fixed cantilever geometry and loop delay used in the simulations of Figure 2. 

The response of the microcantilever in a non-Newtonian fluid shown in Figure 3 differs from the response in a Newtonian fluid (in Figure 2). When the elastic modulus of the fluid increases (Figure 3a), the oscillation frequency decreases (as if the spring constant of the cantilever became weaker), with a respective increase in the amplitude of oscillation. Instead, when the viscous modulus of the fluid increases (Figure 3b), the oscillation frequency and amplitude follow a similar trend to that of the purely viscous fluid. However, the case shown in Figure 3b differs from the case shown in Figure 2 due to the presence of a constant elastic modulus ($G^{\prime }=200$ Pa, in the simulations), which is not present in Newtonian fluids. The dashed burgundy line represents the case of purely viscous water, while the red circles in Figure 3a,b represent imposed phase points that make the system almost insensitive to variations of the elastic and viscous modulus, respectively, as will be discussed in Section 4.2.1.

## 3. Results

The experimental results presented in this section were obtained using a CSG30 gold-coated cantilever from NT-MDT. The natural frequency and quality factor in air were measured in open loop (sweeping the excitation frequency) as *f*_0_ = 65.04 kHz and *Q* = 250.

### 3.1. Newtonian Water/Glycerol Solutions

Five different solutions of water and glycerol were experimentally tested: (i) Pure water; (ii) water + 5% Glycerol (*v*/*v*); (iii) water + 10% Glycerol (*v*/*v*); (iv) water + 20% Glycerol (*v*/*v*); and (v) water + 30% Glycerol (*v*/*v*). These solutions have viscosities of 1.005, 1.174, 1.384, 1.988 and 3.003 mPa s at 20 °C, respectively, with associated densities of 998.02, 1011.3, 1024.6, 1051.2 and 1077.8 kg/m^3^ [23]. 

**Figure 4:**
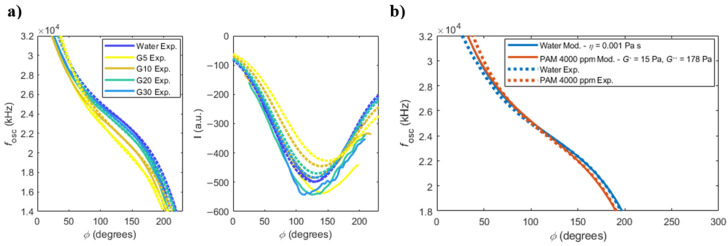
(**a**) Frequency of oscillation and quadrature component of deflection *I*, as a function of the imposed phase in the PLL, for different concentrations of glycerol solutions (solid and dashed lines represent experimental and modelled results, respectively); (**b**) frequency of oscillation, as a function of the imposed phase in the PLL, for water and a viscoelastic solution of 4000 ppm of PAM.

The measured frequency and amplitude responses of the cantilever immersed in the solutions are shown in Figure 4a, as solid lines. The model discussed in Section 2.3 is used and plotted with the dotted lines on top. In the model, Equations (1) and (2) were solved for the frequency and *I* component of deflection, respectively, considering Equations (3)–(5), with *L* = 190 µm, *W* = 30 µm and *T* = 2.5 µm, the values of density and viscosity of each solution, and τ=7.8 µs (measured experimentally from the slope of the phase response of the cantilever in open-loop [32]). A constant ‘gain’ factor between the modelled and experimental curves of *I* is found by dividing the experimental and the modelled curves of water (3.25 × 10^12^). This value is then applied to the other modelled curves in Figure 4a.

The modelled and experimental frequency responses are in good agreement in the central region of the imposed phases in the PLL (from around 70° to 180°), but tend to shift on the edges of the curves. This may be attributed to the loss of effectiveness of the PI-controller in these regions due to the low amplitude of the deflection (see Equation (S10), in the Supplementary Materials, for a description of the transient response of the PI-controller), which causes an offset error.

Conversely, the experimentally measured values of the *I* signal for the different solutions are in disagreement with the modelled curves. According to the model, the *I* signal curves should decrease with increasing viscosity of the glycerol solutions (see also Figure 2). However, this is not what was experimentally observed, as can be seen in Figure 4a. Instead, the measured *I* signal curves appear in a random order, with the *I* signals being unrelated to each other. This problem arises from the lack of precise control of the photothermal excitation mechanism in the current setup, and will be discussed in more detail in Section 4.2.2.

### 3.2. Non-Newtonian Fluid

A solution of 4000 ppm of polyacrylamide (PAM) in water was prepared and used as viscoelastic solution [17,18]. The experimental results of the oscillation frequency of the microcantilever in water and in PAM solution are shown in Figure 4b as dashed lines. The model given by Equation (1), using Equations (6) and (7), is fitted to the experimental results using an elastic and viscous modulus of $G^{\prime }=15$ Pa and $G^{\prime \prime }=178$ Pa, respectively. These values of elastic and viscous modulus agree with the values presented in reference [17] at this range of frequencies. The results of *I* signal are not shown due to the reasons mentioned in the previous section.

## 4. Discussion

### 4.1. Sensitivity of the PLL Platform to Small Changes in Rheological Parameters

#### 4.1.1. Sensitivity to Viscosity Variations in Newtonian Fluids

Equation (1) is highly nonlinear, which prevents a simple analytical estimation of the sensitivity of the PLL system to viscosity variations. However, this sensitivity can be obtained, in a first approximation, by linearising the frequency response of the PLL system with respect to the viscosity, in the form

(8) $$S_{\eta }\left(\phi ,\eta ,\rho ,L,W,\tau ,m_{0},c_{0}\ldots \right)=\frac{\partial f}{\partial \eta }\approx (\frac{f_{2}-f_{1}}{\eta_{2}-\eta_{1}}),$$

where points 1 and 2 define an (ideally small) interval of values of viscosity. Therefore, the sensitivity to viscosity changes can be calculated by measuring the frequency response in two solutions with different viscosities and applying Equation (8). 

**Figure 5:**
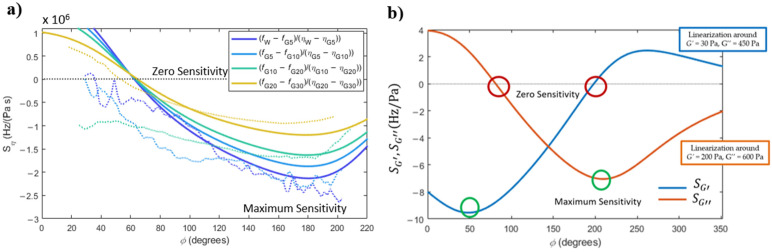
(**a**) Sensitivity to variations in viscosity in a Newtonian fluid (solid and dashed lines represent experimental and modelled results, respectively); (**b**) modelled sensitivity to variations in the elastic and viscous modulus in a non-Newtonian viscoelastic fluid.

Figure 5a shows the modelled results of the sensitivity to viscosity changes as solid lines. Here, Equation (1) was solved (with the same parameters as in Figure 4) considering the values of viscosity of different pairs of solutions, and applying Equation (8). 

Three interesting aspects of the sensitivity curves can be pointed out: (i) as previously described in Figure 2, there is a point of imposed phase in the PLL that makes the system insensitive to viscosity changes (around 65°); (ii) alternatively, the sensitivity to viscosity changes can also be maximised by choosing the appropriate phase imposed in the PLL (around 180°); (iii) finally, the linearised sensitivity, as calculated with Equation (8), is higher for small changes of viscosity (water to G5 solution, for example). 

The dotted lines shown in Figure 5a are sensitivities to viscosity measured experimentally, i.e., calculated from Equation (8) with the experimental frequency curves shown in Figure 4a. Despite the noise due to applying a ‘derivative’ to the experimental curves, all the described trends could be experimentally observed. 

#### 4.1.2. Sensitivity to Variations of Elastic and Viscous Modulus in Non-Newtonian Fluids

An analogous rationale can be used to study the sensitivity of the PLL system to changes in elastic and viscous modulus in non-Newtonian solutions. Considering the elastic and the viscous modulus separately, one can admit that the individual sensitivities are given by

(9) $$S_{G^{\prime }}\left(\phi ,G^{\prime },G^{\prime \prime },\rho ,L,W,\tau ,m_{0},c_{0}\ldots \right)=\frac{\partial f}{\partial G^{\prime }}\approx (\frac{f_{2}-f_{1}}{G_{2}^{\prime }-G_{1}^{\prime }}),$$

(10) $$S_{G^{\prime \prime }}\left(\phi ,G^{\prime },G^{\prime \prime },\rho ,L,W,\tau ,m_{0},c_{0}\ldots \right)=\frac{\partial f}{\partial G^{\prime \prime }}\approx (\frac{f_{2}-f_{1}}{G_{2}^{\prime \prime }-G_{1}^{\prime \prime }}),$$

Figure 5b shows the sensitivities to variations of the elastic and viscous modulus of a non-Newtonian fluid, calculated analytically by applying Equations (9) and (10) to the frequency responses obtained from Equation (1), with the added mass and damping terms given by Equations (6) and (7). The linearised response described by Equations (9) and (10) assume a 2% change of arbitrarily chosen initial conditions: $G^{\prime }=30$ Pa and $G^{\prime \prime }=450$ Pa in the sensitivity to elastic modulus variations, and $G^{\prime }=200$ Pa and $G^{\prime \prime }=600$ Pa in the case of sensitivity to viscous modulus variations.

Some interesting aspects of the curves can still be noted: (i) as previously commented on, zero- and maximum-sensitivity working points can be found, by choosing the appropriate imposed phase in the PLL, for both the elastic and the viscous modulus; (ii) in general, the point of zero sensitivity to the viscous modulus variations corresponds to a point of very high sensitivity of elastic modulus variations, and vice-versa. 

The theoretical results presented here show the possibility of decoupling the effects of the elastic and viscous modulus of viscoelastic solutions.

### 4.2. Inversion Problem to Extract G′ and G″ from the Measured Frequency and Amplitude of the Oscillations in the PLL

#### 4.2.1. Analytical Method

In a PLL platform used for viscoelastic characterisation, one should be able determine the viscous and elastic modulus of the viscoelastic fluid from the measured values of frequency and amplitude of oscillation in the PLL. This requires solving two consecutive steps. 

The first step consists of simultaneously calculating the added mass and damping coefficients, $m_{A}\left(\omega \right)$ and $c_{A}\left(\omega \right)$, due to the non-Newtonian viscoelastic fluid, from the measured values of frequency and normalised amplitude of oscillation. This can be performed using the following equations:

(11) $$m_{A}\left(\omega \right)=\frac{-H\pm \sqrt{H^{2}-4GI}}{2G},$$

(12) $$c_{A}=\frac{EF}{\omega }-Em_{A}\omega -c_{0},$$

where the variables are defined as

$E=-\tan\left(-\frac{\pi }{2}-n\pi +\phi +\omega \tau \right)$, 

$F=m_{0}\left(\omega_{0}^{2}-\omega^{2}\right)$, 

$G=A_{norm}^{2}\omega^{4}\left(1+E^{2}\right)-1$,

$H=-2FA_{norm}^{2}\omega^{2}\left(1+E^{2}\right)-2m_{0}$, 

$I=F^{2}A_{norm}^{2}\left(1+E^{2}\right)-{m_{0}}^{2}$,

These derivations are presented in Part II.1 of the Supplementary Materials. $m_{A}\left(\omega \right)$ and $c_{A}\left(\omega \right)$ are obtained from the negative solution of Equation (11) for the positive part of the variable E (E>0), and vice versa. 

The second step consists of extracting the elastic and viscous modulus, G′ and G″ of the non-Newtonian viscoelastic fluid from the previously calculated $m_{A}\left(\omega \right)$ and $c_{A}\left(\omega \right)$ terms. These calculations were first presented in [16,17,18], and use the following equations:

(13) $$G^{\prime \prime }=\frac{c_{A}\omega }{D}-\frac{B\omega \sqrt{\omega }}{D\sqrt{2D}}{\left[\sqrt{{\left(\frac{B^{2}\omega }{D}+2\left(m_{A}\omega -C\omega \right)\right)}^{2}+4{c_{A}}^{2}}-\frac{B^{2}\omega }{D}-2\left(m_{A}\omega -C\omega \right)\right]}^{\frac{1}{2}}$$

(14) $$G^{\prime }=\frac{\omega^{2}}{D}\left(m_{A}-C-\left(\frac{B^{2}G^{\prime \prime }}{c_{A}\omega -DG^{\prime \prime }}\right)\right),$$

where the variables B, C and D are as defined previously. These derivations are presented in Part II.2 of the Supplementary Materials.

**Figure 6:**
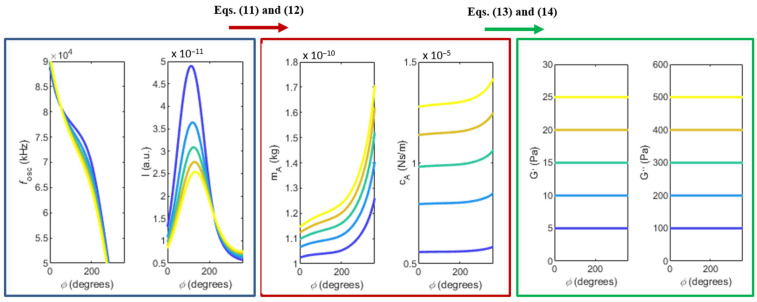
Inversion problem: the frequency and normalised amplitude should be experimentally measured, as indicated in the left panel (these curves were analytically built, instead). From these curves, the added mass and damping terms are calculated, as plotted in the middle panel. Finally, the viscous and elastic modulus of the fluid can be determined, as shown in the right panel.

An example of the complete inversion problem is illustrated in Figure 6, for the sake of clarity. In summary, the idea is to measure the frequency and normalised amplitude of oscillation in the PLL, as shown in the left panel. From the values of frequency and amplitude of oscillation, one should use Equations (11) and (12) to calculate the added mass and damping terms, as shown in the middle panel. Finally, Equations (13) and (14) make it possible to determine the viscous and elastic modulus of the viscoelastic solutions, as shown in the right panel. 

Please note that the curves of frequency and amplitude of oscillation shown in Figure 6 were analytically built instead of experimentally measured. The reasons for this will be discussed in the next section. The simulations leading to the results shown in the left panel of Figure 6 consider the same geometry and loop delay previously used in Figure 3, but with five distinct pairs of viscous and elastic modulus, $G^{\prime }=\left[5,\;10,\;15,\;20,\;25\right]$ Pa and $G^{\prime \prime }=\left[100,\;200,\;300,\;400,\;500\right]$ Pa, assumed to be constant in this range of frequencies for the sake of convenience. Please note that the elastic and viscous modulus (imposed in the model) are recovered in the end of the proposed method, as shown in the right panel of Figure 6. 

#### 4.2.2. Issues to Implement the Inversion Method

Using the current PLL setup, it is still not possible to fully implement the inversion method. As discussed, this method requires calculation of the added mass and damping terms from the measured frequency and normalised amplitude of oscillation (see Equations (11) and (12) and Figure 6). The problem of normalising the amplitude curves (to avoid the unknown constants and gains in the transfer function) can easily be solved by calibrating the amplitude response of the system with a common fluid, such as water. 

The problem to be solved is how to guarantee that the excitation force is constant from experiment to experiment, in order to be able to compare the results of different solutions (this problem was already mentioned when describing the experimental results plotted in Figure 4a). This is due to the fact that, for each experiment (each different solution), the excitation and detection laser are inevitably focused on different points on the cantilever, causing different gains in excitation and transduction [28]. In addition, the optical and thermal properties of each solution change, even if slightly, and this causes differences in the effectiveness of the thermal excitation mechanism and heat losses. All these intricate factors contribute to the observed randomness of the amplitude responses. This is also the reason the curves of frequency and amplitude of oscillation shown in the left panel of Figure 6 were simulated, instead of experimentally measured. As stated, the experimental randomness of the amplitude curves prevents solving the system of two equations comprised of Equations (11) and (12), which is required to use the complete method.

Further modification of the optomechanical configuration and liquid cell are required in order to make it possible to achieve more stable amplitude signals and to use the full proposed method in future experiments.

## 5. Conclusions

A new digital Phase-Locked Loop (PLL) platform, containing a PI-controlled microcantilever, was implemented and used for viscosity and viscoelasticity sensing of Newtonian and non-Newtonian fluids. In this PLL, the user can impose the desired phase between the direct force to the cantilever and the reference demodulationg signals. In addition, the cantilever is photothermally excited, making it possible to obtain a clean and linear response, typical of the forced and damped harmonic resonator. 

Therefore, analytical formulations of the hydrodynamic force caused by the fluid flow around the cantilever can be used to model the dynamical response (frequency and amplitude) of the PLL platform, as a function of the rheological properties of viscous and viscoelastic fluids. These models were validated on the basis of experiments using glycerol and polyacrylamide solutions.

The sensitivity of the system to small variations in the rheological parameters of the solutions was subsequently studied. It was demonstrated that this sensitivity is dependent on the phase imposed in the system, and that working points with zero or maximum sensitivities can be obtained simply by the appropriate choice of the phase value.

For a fixed phase, the PLL can then work in real-time and with very low noise, while detecting changes in the surrounding fluids. The platform can be used to continuously monitor processes, chemical reactions, or to advance fields such as rheokinetics [27], making it possible to overcome most of the limitations of the current open-loop configurations.

## Data Availability

The data presented in this study are available on request from the corresponding author.

## Supplementary Materials

The following supporting information can be downloaded at: https://www.mdpi.com/article/10.3390/s22218421/s1, Part I. Derivation of the general working equations of the PLL, I.1. Added mass and damping in a purely viscous fluid, I.2. Added mass and damping in a viscoelastic Maxwell fluid, Part II. Inversion Problem – Extracting ***G***′ and ***G***″ from the measured frequency and amplitude of the oscillations in the PLL, II.1. Determining ***m_A_*** and ***c_A_*** from the oscillation frequency and amplitude of the PLL, II.2. Determining ***G***′ and ***G***″ from the calculated ***m_A_*** and ***c_A_***, References, Figure S1: Schematic of the electrical signals through the developed PLL platform.
