# Supplementary material for: Photothermal Self-Excitation of a Phase-Controlled Microcantilever for Viscosity or Viscoelasticity Sensing
Source: Sensors (Basel). 2022 Nov 2;22(21):8421. doi: 10.3390/s22218421 (PMC9659050; doi:10.3390/s22218421)
Supplement: Supplementary file 1 [file sensors-22-08421-s001.zip › sensors-1998087-supplementary.pdf]

Part I. Derivation of the general working equations of the PLL

Figure S1 represents a schematic of the electrical signals throughout the PLL platform, and will be used to derive its general working conditions.

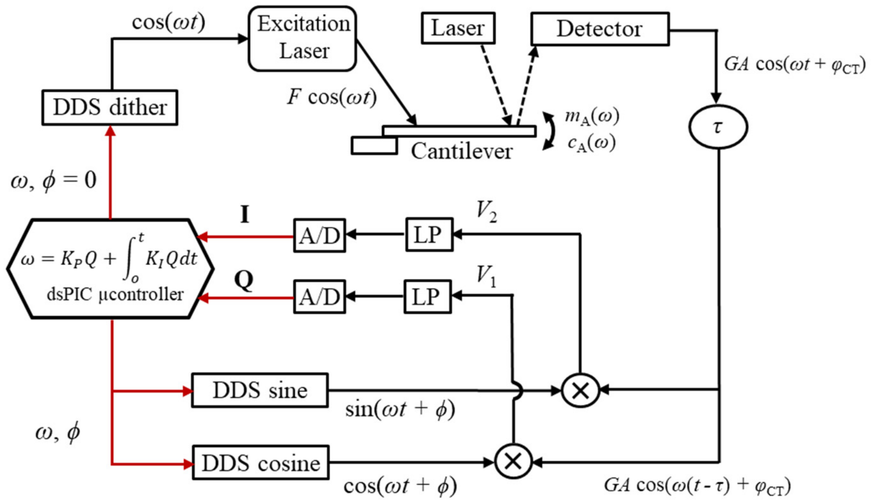

Figure S1. Schematic of the electrical signals through the developed PLL platform.

1. Frequency of oscillation of the PLL, ω

Multiplying the deflection signal by the demodulating cosine reference signal, one gets the signal V_1. After the LP filter, the (digital) DC signal for the in-phase component of the deflection Q is obtained

$$Q = \frac{GA}{2}(\cos\theta\cos\phi + \sin\theta\sin\phi) = \frac{GA}{2}\cos(\theta-\phi), \quad \text{(S1)}$$

where $\theta = -\omega\tau + \varphi_{CT}$, ϕ is the imposed phase in the PLL, F is the magnitude of the force provided by the laser, G is a general transduction gain, A and φ_{CT} are the amplitude and phase of the cantilever and τ represents the total time delay of the signals around the loop, mostly due to the transformation of optical energy into mechanical energy.

Assuming a well-tuned controller, the steady-state of the system will have a null error parameter, or $Q = 0$, which implies the following phase condition

$$\cos(\theta-\phi) = 0 \Rightarrow -\omega\tau + \varphi_{CT} - \phi = -\left(\frac{\pi}{2} + n\pi\right), \text{ with } n = 0, 1, 2, \ldots \quad \text{(S2)}$$

The phase of the oscillating cantilever is given by the transfer function of the forced and damped harmonic oscillator, as $\varphi_{CT} = \text{atan}\left(-\frac{\omega_R\,\omega}{Q_R(\omega_R^2-\omega^2)}\right)$. The resonance frequency and quality factor in viscous fluids were derived by Sader [1] as $\omega_R = \omega_0\left(1+\frac{m_A}{m_0}\right)^{-\frac{1}{2}}$ and $Q_R = \omega_R\left(\frac{m_0+m_A}{c_0+c_A}\right)$. These expressions are introduced in the expression for the cantilever phase, to obtain

$$\varphi_{CT} = \text{atan}\left(-\frac{\omega(c_0+c_A)}{m_0\omega_0^2-(m_0+m_A)\omega^2}\right), \quad \text{(S3)}$$

with ω_0, m_0 and c_0 the natural frequency in vacuum, total mass and intrinsic damping of the cantilever, respectively, and m_A and c_A the added mass and damping coefficients, due to the presence of the surrounding fluid. The phase condition of equation (S2) can finally be written as

$$-\omega\tau + \text{atan}\left(-\frac{\omega(c_0+c_A)}{m_0\omega_0^2 - (m_0+m_A)\omega^2}\right) - \phi = -\left(\frac{\pi}{2} + n\pi\right), \text{ with } n = 0, 1, 2, \dots \quad \text{(S4)}$$

This equation is numerically solved for ω as a function of the imposed phase in the system ϕ.

2. Quadrature Signal, I

Multiplying the deflection signal by the demodulating sine reference signal, one gets the signal V_2. After the LP filter, the (digital) DC signal for the quadrature component of the deflection I is obtained

$$I = \frac{GA}{2}(\cos\theta \sin\phi - \sin\theta \cos\phi) = \frac{GA}{2}\sin(\phi - \theta) = \frac{GA}{2}\sin(-\beta), \quad \text{(S5)}$$

where $\beta = -\phi + \theta$. The frequency condition $Q = 0$ of equation (S2) implies that

$$\cos(\theta - \phi) = 0 \Rightarrow \cos(\beta) = 0 \Rightarrow \beta = -\left(\frac{\pi}{2} + n\pi\right), \text{ with } n = 0, 1, 2, \dots$$

And therefore, I is given by

$$I = \frac{GA}{2}\sin\left(\frac{\pi}{2} + n\pi\right) = \begin{cases} \frac{GA}{2}, \text{ for } n = 0, 2, 4, \dots \\ -\frac{GA}{2}, \text{ for } n = 1, 3, 5, \dots \end{cases} \quad \text{(S6)}$$

3. Amplitude of oscillation, A

The amplitude of oscillation can be obtained from the transfer function of the forced and damped harmonic oscillator, as

$$A = \frac{F}{Gk}\left[\left(\frac{\omega_R^2 - \omega^2}{\omega_R^2}\right)^2 - \left(\frac{1}{Q_R}\frac{\omega}{\omega_R}\right)^2\right]^{-\frac{1}{2}}, \quad \text{(S7)}$$

where $k = m_0\omega_0^2$ is the spring constant of the system. Substituting again the oscillation frequency and quality factor in viscous fluids, as derived by Sader [1], $\omega_R = \omega_0\left(1 + \frac{m_A}{m_0}\right)^{-\frac{1}{2}}$ and $Q_R = \omega_R\left(\frac{m_0+m_A}{c_0+c_A}\right)$, one gets

$$A = \frac{F}{Gm_0}\left[\left(\frac{m_0\omega_0^2}{(m_0+m_A)} - \omega^2\right)^2 + \left(\frac{\omega(c_0+c_A)}{(m_0+m_A)}\right)^2\right]^{-\frac{1}{2}}. \quad \text{(S8)}$$

This amplitude of oscillation can still be normalised, as

$$A_{norm}(\omega) = \frac{(m_0+m_A)}{\left[\left(m_0\omega_0^2 - \omega^2 m_0 - \omega^2 m_A\right)^2 + \omega^2(c_0+c_A)^2\right]^{\frac{1}{2}}}, \quad \text{(S9)}$$

with $A_{norm}(\omega) = \frac{AGm_0}{F}$.

4. Transient response of the PLL

The frequency of oscillation of the PLL is continuously set by the PI-controller implemented in the dsPIC microcontroller, by

$$\omega = K_P Q + \int_o^t K_I Q dt = K_P \frac{GA}{2}\cos(\theta - \phi) + \int_o^t K_I \frac{GA}{2}\cos(\theta - \phi)\, dt, \tag{S10}$$

where the in-phase component Q is used as the error parameter in the PI-controller, with proportional and integral gains of K_P and K_I, respectively. As seen in equation (S10), the transient response of the controller depends on the transducer gain and the amplitude of oscillation.

I.1. Added mass and damping in a purely viscous fluid

The added mass and damping terms, m_A and c_A, of a purely viscous fluid are given by [1], [2]

$$m_{\mathrm{A}}(\omega) = \frac{\pi}{4}\rho L W^2 \left(a_1 + \frac{a_2}{W}\sqrt{\frac{2\eta}{\rho\omega}}\right), \tag{S11}$$

$$c_{\mathrm{A}}(\omega) = \frac{\pi}{4}\rho L W^2 \omega \left(\frac{b_1}{W}\sqrt{\frac{2\eta}{\rho\omega}} + \frac{b_2}{W^2}\frac{2\eta}{\rho\omega}\right), \tag{S12}$$

where ω is the oscillation frequency of the system, ρ and η are the density and viscosity of the fluid, a_1 = 1.0553, a_2 = 3.7997, b_1 = 3.8018, and b_2 = 2.7364 are constants to describe the hydrodynamic function, and L and W are the length and width of the microcantilever.

These terms can be substituted in equations (S4) and (S9) to determine the frequency and amplitude of oscillation of the cantilever in the PLL, immersed in a purely viscous fluid.

I.2. Added mass and damping in a viscoelastic Maxwell fluid

A viscoelastic linear fluid is characterised by a complex dynamic modulus given by $G^* = G' + jG''$, defined by dividing the applied stress by the total strain of the system. The complex modulus contains an elastic part G' and a viscous part G'', with the phase lag between the shear stress and the shear strain given by $\varphi = \arctan\left(\frac{G''}{G'}\right)$. The dynamic modulus G^* can be used to define a complex dynamic viscosity $\eta^* = \eta' + j\eta''$, in which $\eta' = \frac{G''}{\omega}$ is the viscous part and $\eta'' = \frac{G'}{\omega}$ is an elastic viscosity [3], [4].

Substituting the viscosity η by the complex viscosity $\eta^* = \eta' + j\eta'' = \frac{G''}{\omega} + j\frac{G'}{\omega}$ in the hydrodynamic function [1], [2], one gets [5], [6]

$$\tau = a_1 + \frac{a_2\sqrt{2}}{W\omega\sqrt{\rho}}\sqrt{G'' - jG'} + j\left[\frac{b_1\sqrt{2}}{W\omega\sqrt{\rho}}\sqrt{G'' - jG'} - j\frac{2b_2}{W^2\omega^2\rho}G' + \frac{2b_2}{W^2\omega^2\rho}G''\right]. \tag{S13}$$

To solve the complex square root, one should use the following identity:

$$\sqrt{z} = \sqrt{a + ib} = \pm\left(\sqrt{\frac{|z| + a}{2}} + j\frac{b}{|b|}\sqrt{\frac{|z| - a}{2}}\right) = \pm\frac{1}{\sqrt{2}}\left(\sqrt{|z| + a} + j\frac{b}{|b|}\sqrt{|z| - a}\right),$$

from where two distinct solutions of equation (S13) are obtained. Both solutions will be presented, but their physical validity will be assessed later using a particular case. Separating the real and imaginary parts of the hydrodynamic function, as in $\tau = \tau_{re} + j\tau_{im}$, one obtains

$$\tau_{re} = a_1 + \frac{2b_2}{W^2\omega^2\rho} G' + \frac{1}{W\omega\sqrt{\rho}} \begin{bmatrix} a_2\sqrt{\sqrt{G'^2+G''^2}+G''}+b_1\sqrt{\sqrt{G'^2+G''^2}-G''} \\ -a_2\sqrt{\sqrt{G'^2+G''^2}+G''}-b_1\sqrt{\sqrt{G'^2+G''^2}-G''} \end{bmatrix}, \tag{S14}$$

$$\tau_{im} = \frac{2b_2}{W^2\omega^2\rho} G'' + \frac{1}{W\omega\sqrt{\rho}} \begin{bmatrix} -a_2\sqrt{\sqrt{G'^2+G''^2}-G''}+b_1\sqrt{\sqrt{G'^2+G''^2}+G''} \\ a_2\sqrt{\sqrt{G'^2+G''^2}-G''}-b_1\sqrt{\sqrt{G'^2+G''^2}+G''} \end{bmatrix}. \tag{S15}$$

Equations (S14) and (S15) can be further simplified by using the following identity:

$$\sqrt{a \pm b} = \sqrt{\frac{a}{2} + \frac{\sqrt{a^2+b^2}}{2}} \pm \sqrt{\frac{a}{2} - \frac{\sqrt{a^2+b^2}}{2}} = \frac{1}{\sqrt{2}}\left(\sqrt{a+\sqrt{a^2+b^2}} \pm \sqrt{a-\sqrt{a^2+b^2}}\right),$$

with $a = \sqrt{G'^2 + G''^2}$ and $b = G''$, to obtain

$$\tau_{re} = a_1 + \frac{2b_2}{W^2\omega^2\rho} G' + \frac{1}{W\omega\sqrt{2\rho}} \begin{bmatrix} (a_2+b_1)\sqrt{\sqrt{G'^2+G''^2}+G'}+(a_2-b_1)\sqrt{\sqrt{G'^2+G''^2}-G'} \\ -(a_2+b_1)\sqrt{\sqrt{G'^2+G''^2}+G'}-(a_2-b_1)\sqrt{\sqrt{G'^2+G''^2}-G'} \end{bmatrix}, \tag{S16}$$

$$\tau_{im} = \frac{2b_2}{W^2\omega^2\rho} G'' + \frac{1}{W\omega\sqrt{2\rho}} \begin{bmatrix} (b_1-a_2)\sqrt{\sqrt{G'^2+G''^2}+G'}+(a_2+b_1)\sqrt{\sqrt{G'^2+G''^2}-G'} \\ -(b_1-a_2)\sqrt{\sqrt{G'^2+G''^2}+G'}-(a_2-b_1)\sqrt{\sqrt{G'^2+G''^2}-G'} \end{bmatrix}. \tag{S17}$$

Considering the case of a purely Newtonian fluid (no elastic modulus, $G' = 0$), equations (S16) and (S17) reduce to

$$\tau_{re} = 1 + \frac{1}{W\omega\sqrt{2\rho}} \begin{bmatrix} 2b_1\sqrt{G''} \\ -2b_1\sqrt{G''} \end{bmatrix}, \tag{S18}$$

$$\tau_{im} = \frac{2b_2}{W^2\omega^2\rho} G'' + \frac{1}{W\omega\sqrt{2\rho}} \begin{bmatrix} 2b_1\sqrt{G''} \\ -2b_1\sqrt{G''} \end{bmatrix}, \tag{S19}$$

where it is already considered that $a_1 = 1.0$ and $a_2 = b_1$. The solution of equations (S18) and (S19) must reduce to purely viscous case, previously derived by Maali [2] for the hydrodynamic force. This allows to rule out the second solution and finally obtain for the real and imaginary parts of the hydrodynamic functions [5], [6]

$$\tau_{re} = 1 + \frac{2b_2}{W^2\omega^2\rho} G' + \frac{2b_1}{W\omega\sqrt{2\rho}} \sqrt{\sqrt{G'^2 + G''^2} + G'}, \tag{S20}$$

$$\tau_{im} = \frac{2b_2}{W^2\omega^2\rho} G'' + \frac{2b_1}{W\omega\sqrt{2\rho}} \sqrt{\sqrt{G'^2 + G''^2} - G'}. \tag{S21}$$

The added mass and damping terms depend on the real and imaginary parts of the hydrodynamic function, as in [1], [2]

$$m_{\mathrm{A}} = \frac{\pi\rho L W^2}{4} \tau_{re}, \tag{S22}$$

$$c_{\mathrm{A}} = \frac{\pi\rho L W^2 \omega}{4} \tau_{im}. \tag{S23}$$

Substituting equations (S20) and (S21) into equations (S22) and (S23), allows to finally obtain

$$m_{\mathrm{A}} = C + D\frac{G'}{\omega^2} + \frac{B}{\omega}\sqrt{\sqrt{G'^2 + G''^2} + G'}, \tag{S24}$$

$$c_A = D\frac{G''}{\omega} + B\sqrt{\sqrt{G'^2 + G''^2} - G'}, \tag{S25}$$

where $B = \frac{b_1 \pi L W \sqrt{\rho}}{2\sqrt{2}}$, $C = \frac{\pi \rho L W^2}{4}$ and $D = \frac{\pi L b_2}{2}$. These terms can be substituted in equations (S4) and (S9) to determine the frequency and amplitude of oscillation of the cantilever in the PLL, immersed in a non-Newtonian viscoelastic fluid.

Part II. Inversion Problem – Extracting G' and G'' from the measured frequency and amplitude of the oscillations in the PLL

In Part I, it was shown how to use the viscous and elastic modulus to predict the frequency and amplitude of oscillation of the microcantilever immersed in a viscoelastic fluid. However, in a PLL platform used for viscoelastic characterisation, one should be able to solve the inverse problem, i.e., extracting the viscous and elastic modulus of the viscoelastic fluid from the measured values of frequency and amplitude of oscillation in the PLL. This requires solving two consecutive steps, discussed below.

II.1. Determining m_A and c_A from the oscillation frequency and amplitude of the PLL

The first step consists of simultaneously calculating the added mass and damping coefficients, m_A and c_A, due to the non-Newtonian viscoelastic fluid, from the measured values of frequency and normalised amplitude of oscillation.

The PLL phase and amplitude conditions (assuming a perfectly tuned controller and the steady-state of the system) are given by equations (S4) and (S9), here re-written as

$$\left(-\frac{\omega(c_0 + c_A)}{m_0\omega_0^2 - (m_0 + m_A)\omega^2}\right) = \tan\left(-\frac{\pi}{2} - n\pi + \phi + \omega\tau\right), \text{ with } n = 0, 1, 2, \ldots \tag{S26}$$

$$A_{norm}^2\left[(m_0(\omega_0^2 - \omega^2) - \omega^2 m_A)^2 + \omega^2(c_0 + c_A)^2\right] = (m_0 + m_A)^2. \tag{S27}$$

The imposed phase in PLL ϕ is known, while the oscillation frequency, ω, and normalised amplitude, A_{norm}, are measured. Therefore, equations (S26) and (S27) correspond to a system of two equations, which can be directly solved to determine m_A and c_A. Defining the variables

$$E = -\tan\left(-\frac{\pi}{2} - n\pi + \phi + \omega\tau\right),$$

$$F = m_0(\omega_0^2 - \omega^2),$$

$$G = A_{norm}^2\omega^4(1 + E^2) - 1,$$

$$H = -2FA_{norm}^2\omega^2(1 + E^2) - 2m_0,$$

$$I = F^2 A_{norm}^2(1 + E^2) - {m_0}^2,$$

Equations (S26) and (S27) can be written as

$$\frac{\omega(c_0 + c_A)}{F - m_A\omega^2} = E, \tag{S28}$$

$${m_A}^2 G + m_A H + I = 0. \tag{S29}$$

The solution is finally given by

$$c_A = \frac{EF}{\omega} - Em_A\omega - c_0, \tag{S30}$$

$$m_A = \frac{-H \pm \sqrt{H^2 - 4GI}}{2G}, \tag{S31}$$

where m_A and c_A are calculated from the negative solution of equation (S31) for the positive part of the variable E ($E > 0$), and vice-versa.

II.2. Determining G' and G'' from the calculated m_A and c_A

The second step consists of extracting the elastic and viscous modulus, G' and G'' of the non-Newtonian viscoelastic fluid from the previously calculated m_A and c_A terms. These calculations were first presented in [5], [6]. Re-writing equations (S24) and (S25) as

$$m_\mathrm{A}\omega^2 = C\omega^2 + DG' + B\omega\sqrt{\sqrt{G'^2 + G''^2} + G'}, \quad \text{(S32)}$$

$$c_\mathrm{A}\omega = DG'' + B\omega\sqrt{\sqrt{G'^2 + G''^2} - G'}, \quad \text{(S33)}$$

Allows to define two new variables
$K' = m_\mathrm{A}\omega^2 - C\omega^2 - DG'$,
$K'' = c_\mathrm{A}\omega - DG''$.
Calculating the difference and product between these new variables, one gets, respectively

$$K''^2 - K'^2 = -2(B\omega)^2 G' = -2(B\omega)^2\left(\frac{m_\mathrm{A}\omega^2 - C\omega^2 - K'}{D}\right), \quad \text{(S34)}$$

$$K''^2K'^2 = (B\omega)^4 G'' = (B\omega)^4\left(\frac{c_\mathrm{A}\omega - K''}{D}\right)^2 \Rightarrow K'^2 = \frac{(B\omega)^4}{D^2}\left(\frac{c_\mathrm{A}\omega - K''}{K''}\right)^2 \Rightarrow K' = \frac{(B\omega)^2}{D}\left(\frac{c_\mathrm{A}\omega - K''}{K''}\right). \quad \text{(S35)}$$

Substituting equation (S35) for K' into equation (S34), one gets an expression that only depends on K''

$$K''^2 - \frac{(B\omega)^4}{D^2}\left(\frac{c_\mathrm{A}\omega - K''}{K''}\right)^2 = -2(B\omega)^2\left(\frac{m_\mathrm{A}\omega^2 - C\omega^2}{D} - \frac{(B\omega)^2}{D^2}\left(\frac{c_\mathrm{A}\omega - K''}{K''}\right)\right). \quad \text{(S36)}$$

Equation (S36) can be rearranged to

$$K''^4 + K''^2\left(\frac{(B\omega)^4}{D^2} + \frac{2(B\omega)^2}{D}(m_\mathrm{A}\omega^2 - C\omega^2)\right) - \frac{(B\omega)^4}{D^2}(c_\mathrm{A}\omega)^2 = 0, \quad \text{(S37)}$$

and solved using the quadratic form, to obtain (positive solution [6])

$$K''^2 = \frac{(B\omega)^2}{2D}\left[\sqrt{\left(\frac{(B\omega)^2}{D} + 2(m_\mathrm{A}\omega^2 - C\omega^2)\right)^2 + 4(c_\mathrm{A}\omega)^2} - \frac{(B\omega)^2}{D} - 2(m_\mathrm{A}\omega^2 - C\omega^2)\right]. \quad \text{(S38)}$$

Rearranging further, and substituting the variable $K'' = c_\mathrm{A}\omega - DG''$, one gets an explicit dependence for G''

$$G'' = \frac{c_\mathrm{A}\omega}{D} - \frac{B\omega\sqrt{\omega}}{D\sqrt{2D}}\left[\sqrt{\left(\frac{B^2\omega}{D} + 2(m_\mathrm{A}\omega - C\omega)\right)^2 + 4c_\mathrm{A}{}^2} - \frac{B^2\omega}{D} - 2(m_\mathrm{A}\omega - C\omega)\right]^{\frac{1}{2}}. \quad \text{(S39)}$$

Finally, substituting equation (S35) into equation (S34), one gets an expression for G' as function of G''

$$G' = \frac{m_\mathrm{A}\omega^2 - C\omega^2}{D} - \frac{(B\omega)^2}{D^2}\left(\frac{c_\mathrm{A}\omega - K''}{K''}\right) = \frac{m_\mathrm{A}\omega^2 - C\omega^2}{D} - \frac{(B\omega)^2}{D^2}\left(\frac{G''}{c_\mathrm{A}\omega - DG''}\right) \Rightarrow G' = \frac{\omega^2}{D}\left(m_\mathrm{A} - C - \left(\frac{B^2G''}{c_\mathrm{A}\omega - DG''}\right)\right). \quad \text{(S40)}$$

References

[1] J. E. Sader, “Frequency response of cantilever beams immersed in viscous fluids with applications to the atomic force microscope,” *J. Appl. Phys.*, vol. 84, no. 1, pp. 64–76, 1998, doi: 10.1063/1.368002.

[2] A. Maali, C. Hurth, R. Boisgard, Ć. Jai, T. Cohen-Bouhacina, and J. P. Aiḿ, “Hydrodynamics of oscillating atomic force microscopy cantilevers in viscous fluids,” *J. Appl. Phys.*, vol. 97, no. 7, 2005, doi: 10.1063/1.1873060.

[3] J. Mouro, R. Pinto, P. Paoletti, and B. Tiribilli, “Microcantilever: Dynamical response for mass sensing and fluid characterization,” *Sensors (Switzerland)*, vol. 21, no. 1, pp. 1–35, 2021, doi: 10.3390/s21010115.

[4] I. Dufour *et al.*, “The microcantilever: A versatile tool for measuring the rheological properties of complex fluids,” *J. Sensors*, vol. 2012, no. 1, 2012, doi: 10.1155/2012/719898.

[5] N. Belmiloud, I. Dufour, L. Nicu, A. Colin, and J. Pistré, “Vibrating microcantilever used as viscometer and microrheometer,” *Proc. IEEE Sensors*, vol. 4, no. 3, pp. 753–756, 2006, doi: 10.1109/ICSENS.2007.355578.

[6] E. Lemaire, B. Caillard, M. Youssry, and I. Dufour, “High-frequency viscoelastic measurements of fluids based on microcantilever sensing: New modeling and experimental issues,” *Sensors Actuators, A Phys.*, vol. 201, pp. 230–240, 2013, doi: 10.1016/j.sna.2013.07.022.
